# Mediation of exciton concentration on magnetic field effects in NPB : Alq_3_-based heterojunction OLEDs

**DOI:** 10.1039/d3ra03608a

**Published:** 2023-08-07

**Authors:** Jiayi Song, Cheng Wang, Yunxia Guan, Xi Bao, Wan Jiao Li, Lijia Chen, Lianbin Niu

**Affiliations:** a College of Physics and Electronic Engineering, Chongqing Normal University Chongqing 401331 People's Republic of China 20130518@cqnu.edu.cn niulb03@126.com

## Abstract

Organic light-emitting diodes (OLEDs) are considered one of the most promising new display technologies owing to their advantages, such as all-solid-state, high color gamut, and wide viewing angle. However, in terms of special fields, the brightness, lifetime, and stability of the devices need further improvement. Therefore, heterojunction devices with different concentrations were prepared to regulate device brightness. The brightness of the bulk heterojunction device is enhanced by 9740 cd m^−2^, with a growth rate of about 26.8%. The impact of various temperatures and various exciton concentrations on the device magneto-conductance (MC) and magneto-electroluminescence (MEL) was investigated. Experimental results demonstrate that the exciton concentration inside the device can be tuned to improve optoelectronic properties and organic magnetic effects. The complex spin mixing process inside the bulk heterojunction device is deeply investigated, which provides a reliable basis for the design of bulk heterojunction devices.

## Abstract 2

Organic light-emitting diodes (OLEDs) are considered one of the most promising new display technologies owing to their advantages, such as all-solid-state, high color gamut, and wide viewing angle.

## Graphical abstract

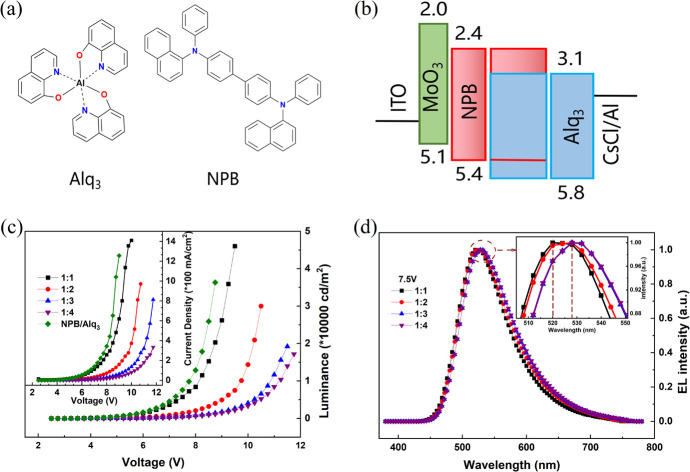

## Introduction

In the past three decades, various properties of OLEDs have attracted considerable attention from researchers in all sorts of aspects, such as operating voltage, brightness, and response speed.^1–5^ In 1987, Tang and VanSlyke prepared organic light-emitting diodes (OLEDs) with low driving voltage using this (8-hydroxy-quinoline)aluminum (Alq_3_) as the light-emitting material.^6^ In 2003, Kalinowski prepared OLEDs using materials without any magnetic properties and found magnetic-field effects (MFEs) in the devices.^7^ In 2010, Lee controlled the magneto-conductance (MC) response of a poly(3-hexylthiophene) (P3HT)-based photovoltaic device by applied magnetic field, bias voltage, built-in potential, and interfacial dipole layers.^8^ MFEs include the magneto-conductive effect and the magneto-electroluminescence (MEL) effect. MC is the change of current with the magnetic field, and MEL is the change of luminous intensity with the magnetic field.^8,9^ In the subsequent studies, researchers began to focus on the influences of external voltage, ambient temperature, functional layer doping, and other factors on the amplitude and line shape of MC and MEL curves.^10–12^ MC and MEL curves are the “fingerprints” of the internal microscopic processes of OLEDs, which visually reflect the role of carriers and provide a method for researchers to study different spin-mixing processes inside the devices.

After decades of research, OLEDs have undergone rapid development and significant improvements in their optoelectronic properties, leading to broad applications in panel displays and lighting applications.^13–15^ However, achieving a high standard of full-color gamut requires further improvement in core indexes, such as brightness, current efficiency, lifetime, and the stability of the devices.^16,17^ In 2015, Xiang *et al.* found in planar heterojunction and bulk heterojunction devices that there was a large difference in linearity between the two at 100 K.^18^ The MEL of planar heterojunction devices decreased because triplet exciplex gathered near the planar heterojunction, prompting the triplet–triplet annihilation (TTA), while the TTA could not occur because there were few triplet exciplexes around the bulk heterojunction. Then Chen *et al.* prepared bulk heterojunction devices of rubrene and C_60_ with MEL curves showing paradoxical voltage dependence, and the analysis discovered that the EL of the half-bandgap voltage devices was not due to the formation of single excitons, originating from TTA in rubrene films.^19^ Recently, Yuan *et al.* improved the electroluminescence performance of exciplex by doping organic spacers into the emitter of bulk heterojunction exciplex; this method achieved low driving voltage, high efficiency, and inefficient attenuation.^20^ To date, most of the studies were focused on the bulk heterojunction solar cells, which exhibit low cost and high energy efficiency, and how to apply their advantages to OLEDs is worth further exploration.^21,22^

**Fig. 1:**
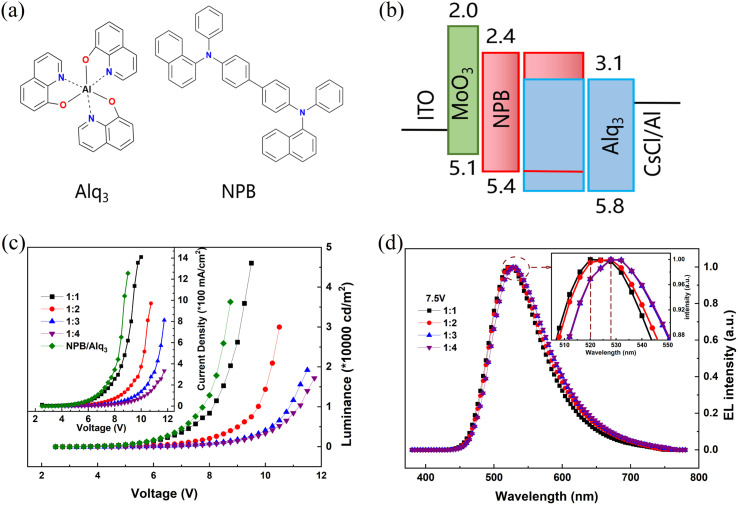
(a) Molecular structure of Alq_3_ and NPB. (b) The energy diagram of the device. (c) Voltage–brightness and voltage–current density curves. (d) Normalized electroluminescence spectra.

In this study, heterojunction OLEDs are prepared by vacuum evaporation, using a P-type semiconductor material *N*,*N*′-di(naphthalene-1-yl)-*N*,*N*′-diphenyl-benzidine (NPB) [reference to the chemical structure in Fig. 1(a)] and an N-type semiconductor material tris(8-hydroxyquinoline)aluminum (Alq_3_) [reference to the chemical structure in Fig. 1(a)]. Fig. 1(b) is the schematic diagram of the device structure energy level diagram of ITO/MoO_3_/NPB/NPB : Alq_3_/Alq_3_/CsCl/Al. We also prepared ITO/MoO_3_/NPB/Alq_3_/CsCl/Al in contrast to the heterojunction devices.

## Results and discussion

In the solid state, there are two primary modes of contact between the two organic semiconductor materials: interlayer contact, which results in a planar heterojunction with minimal material overlap; and intermixing, which leads to an intrinsic heterojunction with significantly greater material overlap than a planar heterojunction.

The materials constituting planar PN heterojunctions mainly include two categories of P-type and N-type materials. The requirements for P-type materials include: (1) having a suitable highest occupied molecular orbital (HOMO) to facilitate hole injection from the anode into the P-type material; and (2) a suitable hole mobility to enable hole transport from the anode interface to the heterojunction interface. The requirements for N-type materials include: (1) a suitable minimum unoccupied molecular orbital (LUMO) for electron injection from the cathode into the N-type material; and (2) a suitable electron mobility for electron transport from the cathode interface to the heterojunction interface. NPB, as a P-type semiconductor material, has a HOMO energy level of 5.4 eV and a high hole mobility (3 × 10^−4^ cm^2^ V^−1^ s^−1^). Alq_3_, as an N-type semiconductor material, has a LUMO energy level of 3.1 eV and a high electron mobility (11.4 × 10^−6^ cm^2^ V^−1^ s^−1^). Therefore, the contact between NPB and Alq_3_ results in the formation of planar PN heterojunction and intrinsic PN heterojunction.^38–40^

The voltage–brightness and voltage–current density curves are shown in Fig. 1(c). Heterojunction devices with NPB : Alq_3_ = 1 : 1 have higher luminance than comparison devices, indicating that the heterojunction can improve the brightness. The maximum luminance of 1 : 1 is 46 040 cd m^−2^, when the voltage is 9 V. However, the maximum reduction drops sharply when the doping ratio changes from 1 : 1 to 1 : 4, and decreases to 17 210 cd m^−2^. The electron mobility of Alq_3_ in the device is 11.4 × 10^−6^ cm^2^ V^−1^ s^−1^, and the hole mobility of NPB is 3 × 10^−4^ cm^2^ V^−1^ s^−1^, so the hole carriers in the devices are the majority carriers. In theory, as the guest Alq_3_ doping concentration increases, the hole concentration in the emitting layer (EML) decreases, electron concentration increases, electron and hole combination probability increases, and device luminance increases. However, in the experiment, as the guest Alq_3_ doping concentration increases, the luminance of the device does not increase rather it decreases. To investigate this interesting phenomenon, we measured the MEL curves of the devices and analyze the energy transfer of the devices. As shown in Fig. 4(b), as the Alq_3_ doping concentration increases, the reduced energy transfer process [Förster energy transfer (FRET) and Dexter energy transfer (DET)] leads to a decrease in the number of singlet excitons (S_1,__Alq3_), and thus the luminescence of the device is diminished. Meanwhile, the decrease in the number of triplet excitons (T_1,__Alq3_) leads to more S_1,__Alq3_ excitons being converted to T_1,__Alq3_ excitons through the singlet fission (SF) process (S_1,__Alq3_ + S_0_ → T_1,__Alq3_ + T_1,__Alq3_), which further reduces the luminance of the device. In addition, the increase of Alq_3_ doping concentration makes the Alq_3_ molecular spacing decrease, which promotes the SF process in the device. In summary, the device maximum luminance decreases as the Alq_3_ doping concentration increases. In the following text, this is analyzed more specifically.

At the same voltage, the current density of the four devices decreases when the doping ratio changes from 1 : 1 to 1 : 4. For example, at 9 V, the current density of devices with NPB : Alq_3_ = 1 : 1, 1 : 2, 1 : 3, and 1 : 4 are about 632 mA cm^−2^, 166 mA cm^−2^, 65 mA cm^−2^, and 35 mA cm^−2^, respectively. Inside the devices, electrons and holes form a compound current *I* in the organic layer, and the total current *I* of the device can be expressed as:^42^1
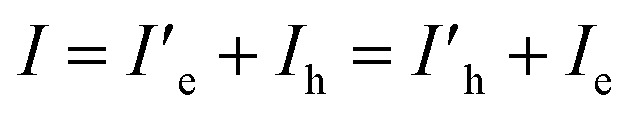
where 
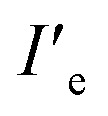
 and 
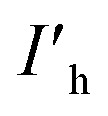
 are leakage currents of electron and hole in the devices, respectively, and *I*_e_ and *I*_h_ are currents *I* of electron and hole in devices, respectively. The electron mobility of Alq_3_ is much smaller than the hole mobility of NPB. Therefore, at the same voltage, as the Alq_3_ doping concentration increases, the electron current *I*_e_ in the device increases slightly and the hole current *I*_h_ decreases significantly, resulting in a lower total device current and making the current density of the device lower.

Fig. 1(d) shows the normalized electroluminescence spectra of the devices at room temperature under a bias voltage of 7.5 V, with the luminescence peak of 520 nm for the 1 : 1 device and 528 nm for the 1 : 2, 1 : 3, and 1 : 4 devices. It indicates that the energy of the doped main body NPB is completely transferred to the guest Alq_3_, NPB does not contribute to the luminescence, and the exciplex is not generated in the devices. With the growth of Alq_3_ doping concentration, the luminescence peak has a slight red shift, which is attributed to the aggregation of Alq_3_ molecules in the luminescent layer.^23^

**Fig. 2:**
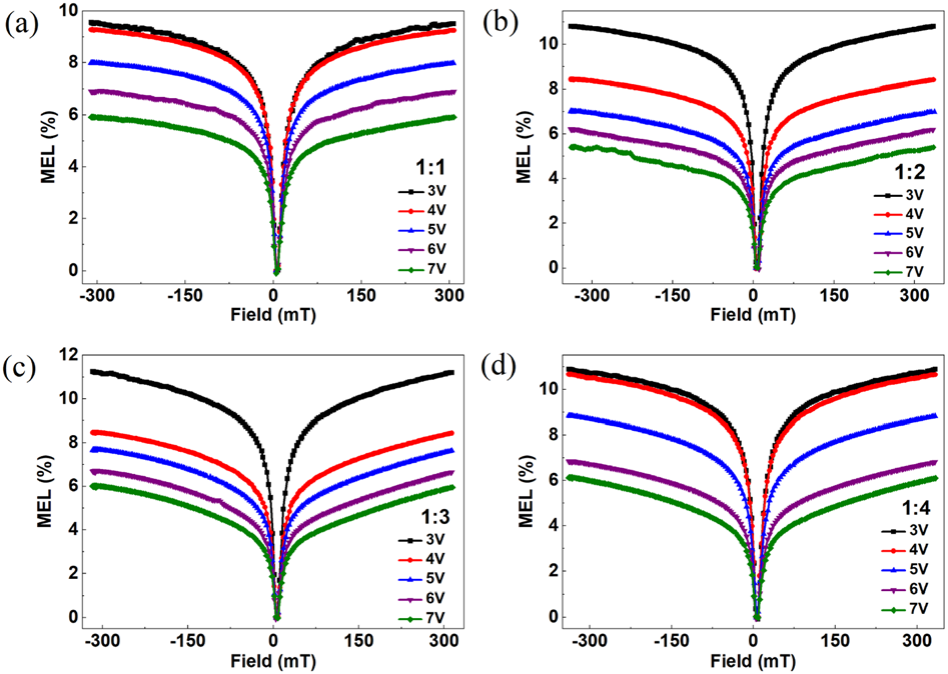
MEL curves of four devices with applied voltage at room temperature (a) device with NPB : Alq_3_ = 1 : 1, (b) device with NPB : Alq_3_ = 1 : 2, (c) device with NPB : Alq_3_ = 1 : 3, and (d) device with NPB : Alq_3_ = 1 : 4.

In considering the impact of external voltage on the magnetic effect of heterojunction devices, Fig. 2 shows the MEL curved line of four devices at different voltages. The definition of MEL is2
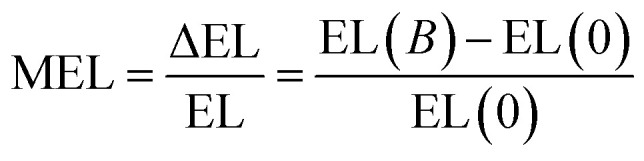
where EL(*B*) and EL(0) represent the brightness of the device with and without an external magnetic field, respectively. From Fig. 2, it can be seen that the MEL curves all show the same trend of change. With Alq_3_ concentration increases, the MEL linearity of the four devices also did not change, that is, the magnetic field is 0–25 mT when the MEL curve increases rapidly, and the magnetic field is 25–330 mT when the MEL curve increases slowly.

To better understand the micromechanical evolution of MEL reflection in devices, the microscopic mechanism of the device is shown in Fig. 4. In organic electroluminescent devices, holes and electrons can be compounded in the NPB : Alq_3_ layer to form polaron pairs. Since there are two spin directions of electrons and holes, there are also two types of polaron pairs, namely, singlet polaron pair (PP_S_) and triplet polaron pair (PP_T_).^10^ Under the hyperfine interaction (HFI),^24,25^ PPs and PP_T_ will undergo spin-mixing and transform into each other (PP_S_ ↔ PP_T_), which is called inter-system crossing (ISC) induced by HFI [Fig. 4(a)].^26,27^ When the magnetic field is applied, PP_T_ becomes PP_T+_, PP_T0_, and PP_T−_ by Zeeman splitting; however, the power of the PP_T0_ state is similar to PPs and is converted to one another (PP_T0_ ↔ PP_S_), this suppresses PP_S_ → PP_T_, causing an increase in the amount of PP_S_.^8^ This inhibition will reach saturation within a few mT.^24,28^ The Coulomb interaction leads to the further conversion of PP_S_ and PP_T_ into singlet exciton (S_1,__Alq3_) and triplet exciton (T_1,__Alq3_). S_1,__Alq3_ is capable of directly radiating prompt fluorescence (PF). At room temperature, T_1,__Alq3_ can not radiate luminescence directly due to the forbidden transition.

For NPB : Alq_3_, it can be observed from Fig. 1(b) that the highest occupied molecular orbital (HOMO) of the doped guest Alq_3_ is higher than that of NPB, and its lowest unoccupied molecular orbital (LUMO) is higher than the LUMO energy level of NPB, so no carrier trap is formed. In addition, the Förster energy transfer (FRET) and Dexter energy transfer (DET) processes are very obvious in host–guest doped devices. FRET can be achieved at distances of a few nanometers due to the need for charge Coulomb interaction, which occurs mainly between the host and guest S_1,__Alq3_ excitons. For FRET, the rate constant (*K*_FRET_) is proportional to the overlapping integral of the PL spectrum of the host material NPB and the absorption spectrum of the guest material Alq_3_. According to the literature,^12,41^ the overlapping area of the absorption spectrum of guest Alq_3_ and the PL spectrum of host NPB suggests that FRET can effectively occur in NPB : Alq_3_ devices. The DET between host and guest T_1,__Alq3_ excitons can be achieved at a distance of a few angstroms by hopping electron and hole transport between molecules. The triplet state energy level of the host material NPB (*E*(T_1,_NPB) = 2.32 eV) is higher than that of the guest material Alq_3_ (*E*(T_1,__Alq3_) = 2.05 eV),^12^ and the Dexter energy transfer (DET) between the host and the guest can be effectively generated in devices with NPB : Alq_3_. As shown in Fig. 4, the formed PP_S_ and PP_T_ consist of two parts, one is formed on the host material and the other is formed on the guest material. S_1,__Alq3_ and T_1,__Alq3_ on the host material are formed on the guest material by FRET and DET, respectively.^29^

**Fig. 3:**
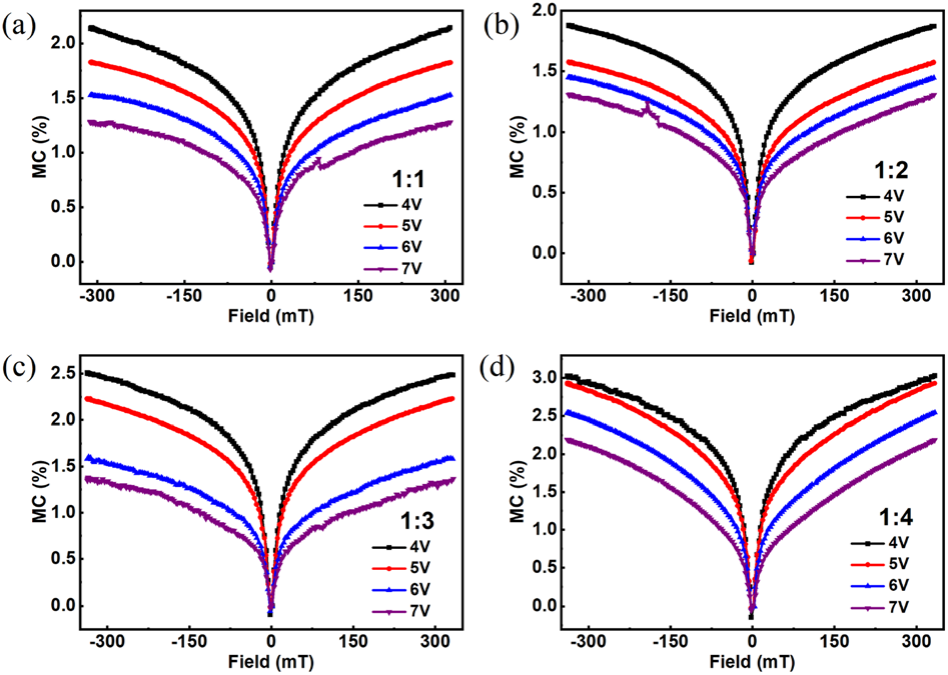
MC curves of four devices with applied voltage at room temperature (a) device with NPB : Alq_3_ = 1 : 1, (b) device with NPB : Alq_3_ = 1 : 2, (c) device with NPB : Alq_3_ = 1 : 3, and (d) device with NPB : Alq_3_ = 1 : 4.

Fig. 2 shows that at the low magnetic field the MEL curve rises rapidly because magnetic field *B* inhibits the ISC process of HFI generation, which increases the S_1,__Alq3_ concentration and the luminescence brightness. As the magnetic field continues to increase, the inhibitory effect reaches saturation and the MEL slowly rises and gradually saturates in the range of 25–300 mT. Fig. 2(a) shows that the amplitude of MEL rise with increasing voltage for NPB : Alq_3_ = 1 : 1 (whose magnetic field is the absolute value of 0–25 mT) decreases continuously from 5.2% at 3 V to 2.9% at 8 V. When an external magnetic field is applied, the electrons and holes are dynamically adjusted to the concentration of S_1,__Alq3_ and T_1,__Alq3_ by feeding at specific angular *ω* = *μ*_B_*gB*/ℏ frequencies, where *μ*_B_ denotes the Bohr magnetic moment, *g* denotes the Lund factor, ℏ denotes the approximate Planck constant, and *B* denotes the magnetic field. With increasing voltage and thus current density, leading to an increase in the concentration of triplet excitons and a decrease in the concentration of singlet excitons, there is an increase in voltage and a decrease in MEL amplitude instead. In addition, the MC curves of the four devices at different voltages were also studied, as shown in Fig. 3. The definition of MC is3
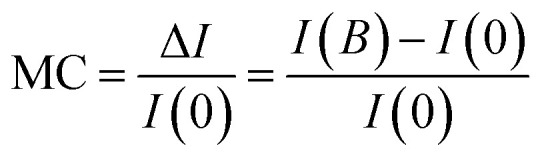
where I(*B*) and I(0) represent the currents of the devices with and without an external magnetic field, respectively. The spin-mixing process of exciton inside the device can be analyzed by MC. Due to the stronger dissociation of PP_S_, it is significantly more likely than PP_T_ to dissociate into free charges and thus enhance the conduction current of the device. This means that the magnetic field suppresses the ISC effect, which contributes to an increase in the amount of PP_S_ and secondary carriers formed by its dissociation, resulting in enhanced MC within a smaller magnetic field range. Similarly, the suppression of the ISC process by the applied magnetic field quickly saturates.^6^ So the MC of Fig. 3 will show a rapid rise from 0 to 25 mT and a slow rise and gradual saturation from 25 to 330 mT.

**Fig. 4:**
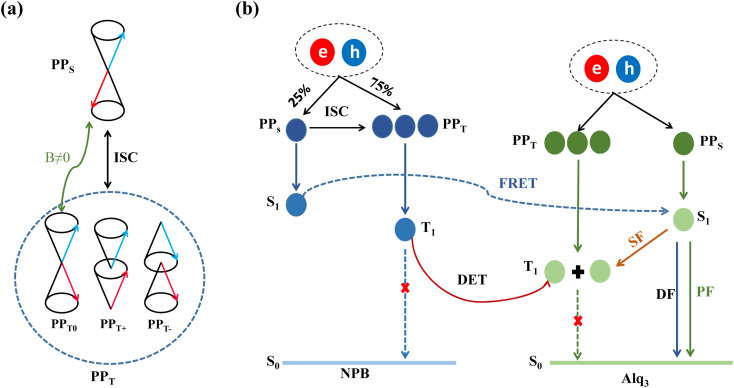
(a) PP_S_ and PP_T_ change process and (b) the schematic of energy transfer and microscopic process in device.

**Fig. 5:**
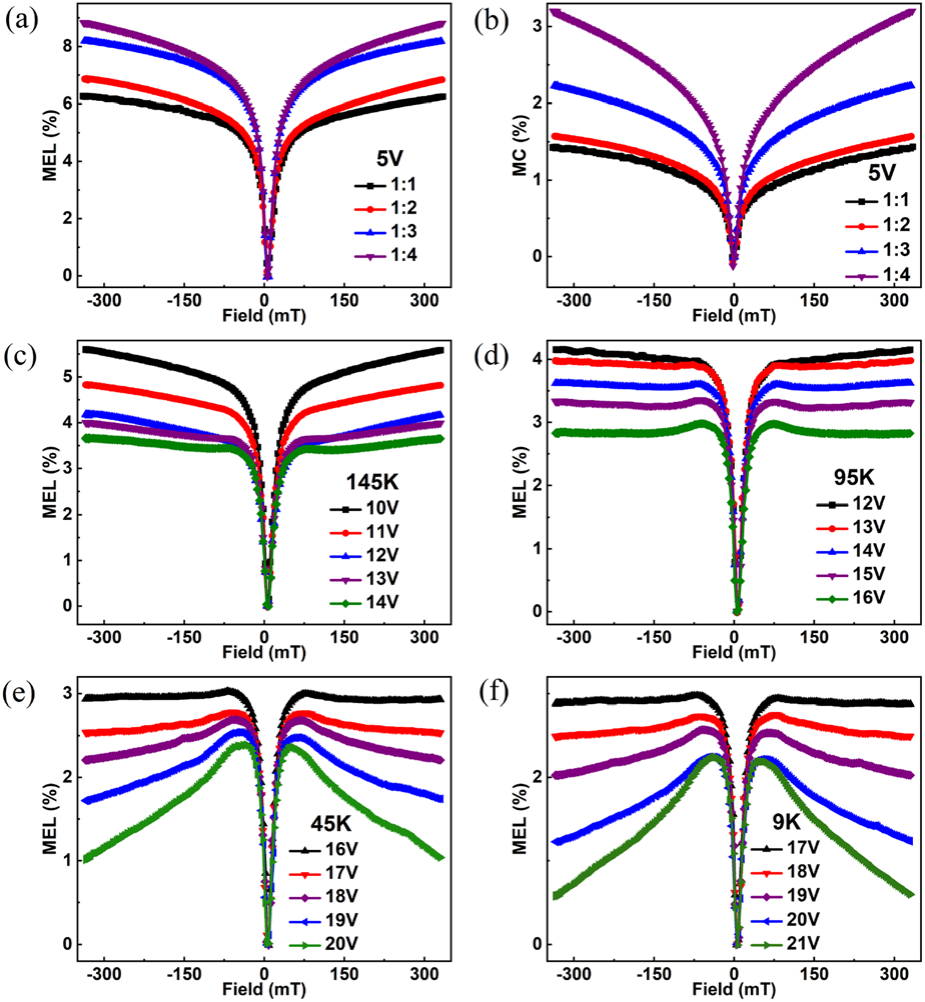
(a) MEL comparison chart of four devices at 5 V, (b) MC comparison chart of four devices at 5 V, (c) MEL comparison chart at 145 K, (d) MEL comparison chart at 95 K, (e) MEL comparison chart at 45 K, and (f) MEL comparison chart at 9 K.

To investigate the influence of exciton concentration on heterojunction devices, the MEL and MC of devices with different Alq_3_ dosages at the same voltage (5 V, for example) are compared, as shown in Fig. 5(a) and (b). MEL and MC in the figure show similar linearity to that in Fig. 2, a fast rise caused by HFI and ISC at low magnetic fields, soon saturated under the high field. However, from Fig. 5(a) and (b) we can see that the amplitude of the MC and MEL curves increase with growing Alq_3_ dosages at 5 V, and both MC and MEL increase by 1.4%, but all are smaller than the MC and MEL amplitude of NPB : Alq_3_ = 1 : 4. The hole mobility of NPB is much larger than the electron mobility of Alq_3_; therefore, the charge is unbalanced. It can be seen from Fig. 1(b) that holes are more easily injected than electrons. When NPB : Alq_3_ = 1 : 1, the amount of NPB in the light-emitting layer increases, and too many holes make the device unbalanced; when NPB : Alq_3_ = 1 : 4, the amount of Alq_3_ in the light-emitting layer increases, and the growth of electrons leads to an increase in the degree of equilibrium of the device. Therefore, from NPB : Alq_3_ = 1 : 1 to NPB : Alq_3_ = 1 : 4, it shows the process of the device from disequilibrium to equilibrium. As shown in Fig. 4(b), the singlet fission (SF) process (S_1,__Alq3_ + S_0_ → T_1,__Alq3_ + T_1,__Alq3_) dominates within the Alq_3_ molecule. As the doping ratio of Alq_3_ increases, resulting in increased SF processes in the luminescent layer, and the phenomenon of increasing amplitude of MC and MEL appears. At the same time, the SF process is enhanced and more S_1,__Alq3_ excitons in the luminescent layer are converted to T_1,__Alq3_ excitons, reducing the prompt fluorescence of the devices. Thus, it can be seen that the exciton dosage has a momentous influence on the organic magnetic effect.

Fig. 5(c)–(f) shows the MEL of NPB : Alq_3_ = 1 : 2 at different voltages and temperatures of 145 K, 95 K, 45 K, and 9 K. When the temperature is 145 K and 95 K, the MEL curves of NPB : Alq_3_ = 1 : 2 are similar to the experimental results of Fig. 2 at room temperature, the MEL curves rise rapidly at the magnetic field of 0–25 mT and rise slowly and saturate at the magnetic field of 25–330 mT. This phenomenon is still caused by the inhibitory effect of HFI and magnetic field on ISC. As temperature decreases further, Fig. 5(e) and (f) exhibit distinct MEL curves from Fig. 5(c) for the magnetic fields of 25–330 mT with no saturation observed but rather a gradual decrease in intensity.

According to the spin-statistics principle, the ratio of PP_S_ to PP_T_ based on spin pairing within organic electroluminescent devices is 1 : 3.^30,31^ In fluorescent devices, S_1,__Alq3_ generates fluorescence, while T_1_ does not contribute directly to the luminescence due to the forbidden transition. It was found that under certain conditions, S_1,__Alq3_ and T_1,__Alq3_ can be interconverted, and two T_1,__Alq3_ can produce an S_1,__Alq3_ and a ground-state molecule, a process known as TTA,^32–35^ creating delayed fluorescence. However, the external magnetic field will have an inhibitory effect on the TTA, weakening the delayed fluorescence and causing the electroluminescence intensity to how a reduced trend in the high magnetic field. The TTA process and the T_1,__Alq3_ exciton lifetime can be expressed by the following equation.4
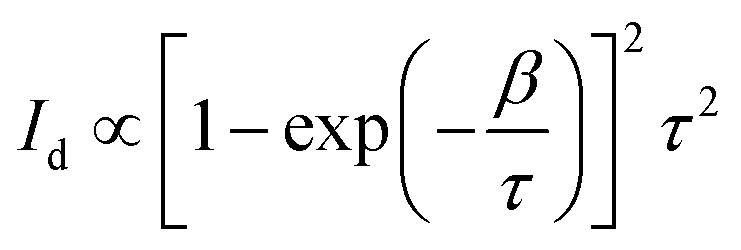
where *I*_d_ denotes the initial intensity of the TTA process, *τ* denotes the T_1,__Alq3_ exciton lifetime, and *β* denotes the constant associated with the pulse width. When the temperature is higher, the T_1,__Alq3_ lifetime is shorter due to the effect of thermal noise, such as phonons, and the TTA process is difficult to occur in the high magnetic field part. When the temperature drops from 145 K to 45 K, the lifetime of T_1,__Alq3_ is extended and the TTA process dominates at this time, but the external magnetic field will inhibit the TTA effect, so the MEL curve at 45 K and 9 K shows a slow decrease in the high magnetic field part,^36,37^ and the temperature is lower, the fall in the high magnetic field part of the MEL is more obvious. In conclusion, the temperature can effectively affect the spin-mixing process inside the device.

## Experimental

Each functional layer material was vaporized on the substrate glass using JD-450 coating equipment, and the functional layer material was presented in the form of a thin film on the substrate glass. Heterojunction devices with the structure of ITO/MoO_3_ (5 nm)/NPB (30 nm)/NPB : Alq_3_ (1 : *x*, 70 nm)/Alq_3_ (40 nm)/CsCl (0.6 nm)/Al (120 nm) [*x* = 1, 2, 3, and 4] were prepared. Before the experiment, ITO substrate glass needs to be pre-processed as follows: first, the ITO surface is scrubbed for 2–3 min to remove impurities, such as oil and dust, from the ITO surface, and the substrate glass is rinsed repeatedly after scrubbing until there is no detergent. Then ITO surface is ultrasonically cleaned with acetone for 30 min, followed by wiping for 5–6 min. After wiping, ITO is ultrasonicated with deionized water, anhydrous ethanol, and acetone for 15 min, dried for 5 min, and ozonated for 30 min. The pretreatment of the ITO substrate can improve surface function. In the experiment, the treated ITO substrate glass was put into the coating equipment, placed the material in the vacuum chamber, and then various functional layer materials were vaporized in turn. During evaporation, the vacuum level was kept at about 10^−4^ Pa to prevent the organic materials from being oxidized by water. In the experiments, a film thickness detector (SI-TM206C) was used to monitor the evaporation rate and film thickness. The device optoelectronic data and magnetic effect data were measured by Janis CCS-350S and LakeShore-643, respectively. A PR-655 spectrometer was used to measure the electroluminescence spectra of the devices.

## Conclusions

In summary, four heterojunction devices with the structures of ITO/MoO_3_ (5 nm)/NPB (30 nm)/NPB : Alq_3_ (1 : *x*, 70 nm)/Alq_3_ (40 nm)/CsCl (0.6 nm)/Al (120 nm) [*x* = 1, 2, 3, and 4] were prepared. Compared with the reference device, the maximum brightness of the heterojunction device is 46 040 cd m^−2^, which is an improvement of 9740 cd m^−2^. It was found that the MC and MEL curve amplitudes decreased with increasing applied bias voltage for the same doping ratio at room temperature and increased with growing Alq_3_ doping concentration for the alike bias voltage. In addition, the impact of temperature on the MEL curve of the device was researched. At 45 K and 9 K, the MEL curves show a different phenomenon from room temperature, the high magnetic field part decreases slowly, which is caused by the TTA that plays a dominant role at low temperature. In this study, the influence of exciton concentration on the magnetic effect of heterojunction device is investigated in depth, and the complex spin mixing process inside the heterojunction device is explored and to provide a data reference for the design of heterojunction devices.

## Author contributions

Jiayi Song: investigation, writing, original draft. Cheng Wang: review & editing, data analysis. Xi Bao: writing & editing. Wanjiao Li: investigation. Lijia Chen: conception. Yunxia Guan: review & editing. Lianbin Niu: supervision, resources.

## Conflicts of interest

There are no conflicts to declare.

## Supplementary Material
